# Metabolic syndrome agravates cardiovascular, oxidative and inflammatory dysfunction during the acute phase of *Trypanosoma cruzi* infection in mice

**DOI:** 10.1038/s41598-019-55363-9

**Published:** 2019-12-11

**Authors:** Bruno Fernando Cruz Lucchetti, Natalia Boaretto, Fernanda Novi Cortegoso Lopes, Aparecida Donizette Malvezi, Maria Isabel Lovo-Martins, Vera Lúcia Hideko Tatakihara, Victor Fattori, Rito Santo Pereira, Waldiceu Aparecido Verri, Eduardo Jose de Almeida Araujo, Phileno Pinge-Filho, Marli Cardoso Martins-Pinge

**Affiliations:** 1 Department of Physiological Sciences, Center of Biological Sciences, State University of Londrina, Londrina, PR Brazil; 2 Department of Pathological Sciences, Center of Biological Sciences, State University of Londrina, Londrina, PR Brazil; 3 Department of Histology, Center of Biological Sciences, State University of Londrina, Londrina, PR Brazil; 4 Department of Physiotherapy, University Center of Araguaia Valley, Barra do Garças, MT Brazil

**Keywords:** Interleukins, Metabolic syndrome, Cardiomyopathies

## Abstract

We evaluated the influence of metabolic syndrome (MS) on acute *Trypanosoma cruzi* infection. Obese Swiss mice, 70 days of age, were subjected to intraperitoneal infection with 5 × 10^2^ trypomastigotes of the Y strain. Cardiovascular, oxidative, inflammatory, and metabolic parameters were evaluated in infected and non-infected mice. We observed higher parasitaemia in the infected obese group (IOG) than in the infected control group (ICG) 13 and 15 days post-infection. All IOG animals died by 19 days post-infection (dpi), whereas 87.5% of the ICG survived to 30 days. Increased plasma nitrite levels in adipose tissue and the aorta were observed in the IOG. Higher INF-^γ^ and MCP-1 concentrations and lower IL-10 concentrations were observed in the IOG compared to those in the ICG. Decreased insulin sensitivity was observed in obese animals, which was accentuated after infection. Higher parasitic loads were found in adipose and hepatic tissue, and increases in oxidative stress in cardiac, hepatic, and adipose tissues were characteristics of the IOG group. Thus, MS exacerbates experimental Chagas disease, resulting in greater damage and decreased survival in infected animals, and might be a warning sign that MS can influence other pathologies.

## Introduction

For more than 100 years, the protozoan *Trypanosoma cruzi* has been known as the causative agent of Chagas disease (CD); however, this disease is still considered a major public health and social problem throughout Latin America. Despite its impact on the morbidity and mortality of affected people, CD remains a neglected tropical disease according to the World Health Organization (WHO)^1^. In 2012, the WHO characterised CD as “the most neglected among neglected diseases”^2^. Further, it is considered endemic in tropical and subtropical areas, distributed throughout the Americas, from the southern US to northern Argentina^3^, and has become an emerging global problem in non-endemic areas. After control vector transmission and transfusional CD, the perpetuation of infection occurs mainly through congenital transmission in endemic and non-endemic areas, whereas in rural areas, oral infection outbreaks are more significant^4^. Worldwide, an estimated 6 to 7 million people are afflicted with CD, causing more than 10,000 deaths per year^5^.

During the acute phase of *T*. *cruzi* infection, the first interaction between the parasite and the host takes place^6^. For the effective control of *T*. *cruzi*, a response involving innate and adaptive immune cells and mainly the production of pro-inflammatory Th1 cytokines such as interferon (IFN)-^γ^, tumour necrosis factor (TNF)-α, and interleukin (IL)-12 is required^7^. However, the increase in these cytokines might lead to undesirable tissue damage in the host caused by increased nitric oxide (NO) production by macrophages^8,9^. It is thus very likely that interactions between immunological events and the parasite upon first contact can significantly change the course of CD with respect to both protective and pathogenic responses in the chronic phase^6^.

Obesity is a major public health concern worldwide, contributing to increased morbidity and mortality. In recent years there has been an accelerated increase in the prevalence of obesity worldwide that has been termed “globesity”^10,11^. Obesity, especially visceral obesity, is strongly associated with inflammatory pathogenic factors that can contribute to the development of insulin resistance, increase the synthesis of low-density lipoproteins, and elevate blood pressure, thus contributing to the development of metabolic syndrome (MS)^12^. MS is a complex disorder that is defined as a set of mutually related factors including abdominal obesity, increased blood pressure, insulin resistance, and dyslipidaemia. These combined factors directly raise the risk of cardiovascular diseases including stroke and type 2 diabetes mellitus^13^.

In recent years, there has been a significant change in the life habits of the Latin population, a region considered endemic for *T*. *cruzi* infection, mainly involving a shift from traditional eating habits to a more westernised diet richer in fat and sugar. Due to this behaviour, the prevalence of obesity and MS in this population has been growing^14^. Considering this, it is necessary to understand the effects of the interaction between MS and CD, especially since *T*. *cruzi* can infect host adipocytes, which can result in changes to their normal function and possibly altering the parasitaemia, tissue parasitic load, and cardiac pathology^15–18^. Accordingly, it is possible that in the acute phase of *T*. *cruzi* infection, MS might exacerbate the deleterious effects of experimental CD.

The MSG model is induced by the subcutaneous administration of monosodium glutamate (MSG) in new-born mice, which results in the development of several metabolic changes, resulting from central hypothalamic effects, during adulthood, promoting changes in peripheral metabolism, among other effects, and deficits in the production of growth hormone. Therefore, MSG animals are smaller and weigh less, but have a hyperadipose phenotype. In addition, the MSG model presents with several other pathophysiological effects that coincide with known changes in MS in humans, such as hypertension, hyperleptinemia, increased abdominal fat, and insulin resistance, among others^19–21^. In addition, this model of obesity induction is widely accepted for the study of MS^22,23^.

## Material and Methods

### Animals

The experimental protocols were performed in accordance with the Guide for the Care and Use of Laboratory Animals and the Ethical Principles for Animal Experimentation established by the Brazilian Committee for Animal Experimentation (COBEA). All procedures and the maintenance of *T*. *cruzi* were approved by the Committee of Ethics and Research of Animals of the State University of Londrina (process number: 19665.2016.03). All animals were housed in polypropylene boxes (414 × 344 × 168 mm). The boxes were lined with autoclaved brush and washed three times per week. The boxes remained in the conditioning room (temperature, 21–23 °C) under a 12-h/12-h light/dark cycle. The animals had free access to water and feed.

### Obesity induction

To induce obesity, new-born Swiss mice underwent subcutaneous injection of MSG (Sigma, St. Louis, USA; 4 mg/g body weight) from day 1 to day 6 after control mice received an equimolar solution of saline injected in animals of the obese group^24^. The mice were separated after weaning by sex and weighed every 10 days; only male mice were used. Obesity was characterised by the Lee Index for each animal using the formula: ∛body weight/naso-anal length × 1000^25^. The abdominal circumference of the mice was also measured, as the weight of retroperitoneal and perigonadal fats.

### Measurement of cardiovascular parameters

Cardiovascular parameters were measured at two timepoints; the first was performed during the development of obesity, when mice were 30, 40, 50, 60, and 70 days of age. The second occurred on days 7, 9, 13, 15, 19, 21, and 30 days post-infection. Measurements were obtained through the non-invasive CODA system (Kent Scientific, Torrington, CT) based on the volume of pressure obtained from the mouse tail^26,27^.

### Infection

When mice were 70 days of age, they were randomly assigned to four groups as follows: control (CG, saline solution and uninfected); infected control group (ICG, received saline solution, infected with *T*. *cruzi*); obese control group (OCG, subjected to obesity induction protocol and uninfected); infected obese group (IOG, subjected to obesity induction protocol via MSG and infected).

For the infected groups, an intraperitoneal inoculum of 5 × 10^2^ trypomastigotes of the Y strain of *T*. *cruzi* was used. In the uninfected groups, a phosphate buffered saline (PBS) control inoculum with the same volume was administered. The Y strain was maintained *in vivo* by inoculations performed every 10 days with 5 × 10² *T*. *cruzi* blood trypomastigotes, diluted in PBS, intraperitoneally in Swiss mice.

### Parasitaemia and survival

Parasitaemia was evaluated in 5 μL of heparinised venous blood obtained from mouse tails. The number of parasites in 50 microscopic fields at 400× magnification was counted using an Olympus CH30LF100 - (Olympus Optical CO., LTD) light microscope. This procedure was performed on days 7, 9, 13, 15, 19, 21, and 30 after infection. The data obtained were expressed as number of parasites per mL^28^. Mouse survival rates were evaluated for 30 days post-infection.

### Collection of organs, tissues and blood

**Figure 1:**
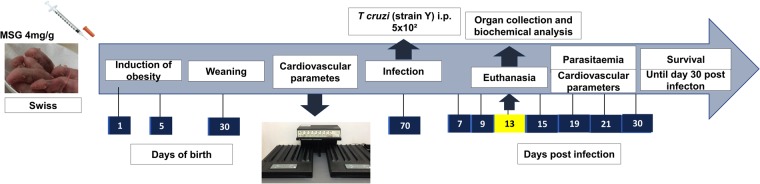
Experimental Design. Swiss mice in first day of life begin treatment with monosodium glutamate (4 mg/g) during five consecutive days. After thirty days the mice was weaning and the male mice was separated. From the 30^th^ to the 70th day of life the mice had the cardiovascular parameters evaluated. On 70^th^ day the mice were infected with 5 × 10² tripomastigotes (Y strain). In the 7^th^ day after infection we started the evaluation of cardiovascular parameters and parasitaemia until day 30 post infection. In 13^th^ day a part of group of mice was euthanized for perform other experiments.

On the thirteenth day after infection, the mice were anaesthetised (100 mg/kg ketamine and 10 mg/kg xylasina) and blood was obtained by cardiac puncture. The blood was transferred to microtubes containing 30 μL of the ethylenediaminotetraacetic acid anticoagulant (Newprov, Pinhais-PR). The blood was centrifuged for 10 min at 2600 × *g*, and plasma was collected and stored at −80 °C until subsequent analyses. After cardiac puncture, the animals were euthanised via cervical dislocation and the retroperitoneal and perigonadal adipose tissue, heart, liver, and aorta were collected. The timepoints of infection and tissue harvesting are shown in Fig. 1.

### Histopathological analysis of the liver

The liver was immediately fixed in 10% buffered formalin. Tissue was embedded in paraffin and three 4-μm sections were obtained per animal. The histological sections were stained with haematoxylin and eosin (HE). Three 5-μm sections were also obtained and stained with periodic acid–Schiff (PAS) to visualise glycogen storage in hepatocytes (purple)^29^.

Hepatic tissue parasitism was evaluated by counting the number of amastigotes based on 20 microscopic fields at 200× magnification by conventional light microscopy (Olympus Model CH30LF100 - Olympus Optical CO., LTD). The results were expressed as mean number of amastigote nests per 20 microscopic fields.

The liver inflammatory infiltrate was evaluated in 10 fields per section of tissue with a light microscope at a magnification of 100×. The inflammation was graded using a classification scale based on four stages as follows: negative or 0 (inflammatory cells in 10 microscopic fields = 0), mild inflammation or 1 (mean inflammatory cells in 10 microscopic fields = 1–10), moderate inflammation or 2 (mean inflammatory cells in 10 microscopic fields = 11–49), severe inflammation or 3 (mean inflammatory cells in 10 fields microscopic ≥ 50)^30^.

Hepatic steatosis was evaluated in the same 10 fields used to evaluate the inflammatory infiltrate. To classify the intensity of steatosis, a scale of score of 0 to 4 was used, taking into account the percentage of hepatocytes containing vacuoles in their cytoplasm as follows: 0 (<5%), 1 (5–20%), 2 (20–30%), 3 (30–60%), 4 (>60%)^31^.

The degree of hepatic glycogen was graded using a positive hepatocyte quantity scale from 0 to 4+ in the centrilobular and perilobular regions.

### Biochemical parameters

On the 13^th^ day after infection, food and water were removed from the animal’s cage for approximately 12 h before blood collection via cardiac puncture. The blood was collected without any anticoagulant, and allowed to coagulate for 60 min at room temperature. After coagulation, samples were centrifuged for 10 min at 2600 × g for serum removal.

Serum levels of total cholesterol, triglycerides, alanine aminotransferase (ALT), and aspartate aminotransferase (AST) were measured using the Dimension RxL Max biochemical analyzer (Siemens, Munich, Germany) commercial kits supplied by the manufacturer.

### Quantification of nitrite in plasma and tissues

Nitrite concentrations in cardiac and aortic tissues and plasma of control and infected mice were assessed on the 13^th^ day after PBS or *T*. *cruzi* inoculation. The concentration of nitrite was estimated as described previously^32^ with some modifications proposed by Panis, *et al*.^33^

### Plasma cytokine levels

IL-6, IL-10, MCP-1, IFN-γ, TNF, and IL-12 were determined with a cytometric bead array (BDTM CBA mouse inflammation Kit, San Jose, CA, USA). Briefly, 50 µL plasma samples were subjected to analysis in duplicate using the cytometric bead array kit with a C5 cytometry. The concentration of plasma cytokines was quantified using FCAP Array^TM^ v. 3.01, SoftFlow©.

### Oxidative stress analysis

On the 13^th^ day after infection, collected tissues were submitted to the following tests: ABTS, which evaluates the total antioxidant capacity through the ability of the of antioxidant molecules to neutralise the radical cation ABTS (ABTS+)^34^; the ability of tissues to reduce iron ions, as determined by the ferric reducing ability of plasma (FRAP) assay^34^; the nitroblue tetrazolium (NBT) assay, which evaluates superoxide anion production based on the reduction of NBT reagent^35^; evaluation of lipid peroxidation, which was determined based on the levels of thiobarbituric acid reactive substances (TBARS)^36^.

### Insulin tolerance test

These tests were performed on the 13th day after infection, after a 4-h morning fast^37,38^. Topical application of lidocaine ointment at 50 mg/g (AstraZeneca do Brasil Ltda - Cotia - SP) at the end of the animal’s tail was performed prior to experiments to reduce pain. After analgesia, 2 μL of blood was collected for baseline blood glucose measurements, which was considered time 0 in the insulin tolerance test. Then, an intraperitoneal injection of human recombinant DNA-derived human insulin solution (ELI LILLY And Company - IN, USA) diluted in sterile PBS was administered. Blood samples were collected at 15, 30, 60, 90, and 120 min after insulin administration. Blood glucose was measured using Accu-Chek Active reactive strips (Roche Diagnostics - IN, USA) that were read on a device from the same manufacturer.

The glucose decay constant (Kitt) was calculated by the formula 0.693 / t1/2, where t1/2 is the half-life of the plasma glucose calculated based on the slope of the curve obtained during the linear phase of plasma glucose decay detected at different times^39,40^. The Kitt value means insulin sensitivity^41^.

### Statistical analysis

The Shapiro-Wilk test was used to test normality of the data. If data were normally distributed, a Student’s t-test was used to compare two groups and a one-way analysis of variance (ANOVA) with Tukey’s post-test was used for more than three groups. For grouped data, we used two-way ANOVA with Tukey’s or Sidak’s post-test. When the data were not normally distributed, a Mann Whitney and Kruskall-Wallis test were used. The values are presented as mean ± SEM. The results were considered significant when p < 0.05. Survival rates were determined by the Gehan-Breslow-Wilcoxon test.

## Results

**Figure 2:**
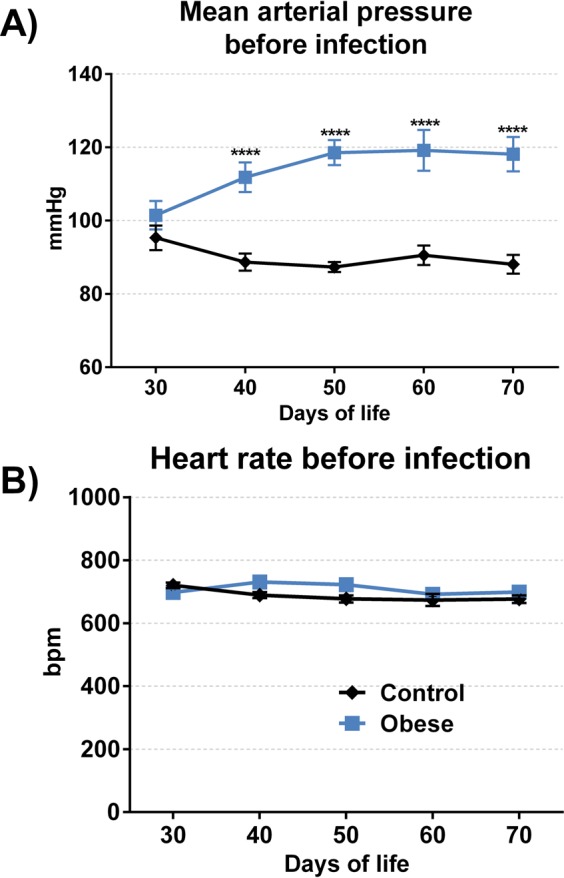
Cardiovascular parameters during development of obesity. The analysis of cardiovascular parameters began in 30^th^ of life of mouse and repeated every 10 days until 70^th^ of life, using the Coda Platform. The mean arterial pressure **(A)** was expressed in millimeters of mercury (mmHg) and the heart rate **(B)** was expressed in beats per minute. Data show mean ± SEM. ****p < 0.0001 when compared control group *vs* obese group. The number of animals used was: 30 control and 23 obese mice.

The animals in the control group weighed more during the whole period of evaluation, and only on the 70th day of age, no statistical difference was found between the body masses of control group (CG) and obese group (OG) mice, **(**Supplementary Data**)**. The characteristic of this model are presented in Table 1. At 30 days of age, both CG and OG mice had similar mean arterial pressure (MAP) values. From day 40, a higher MAP was found in the OG when compared to that in the CG, which persisted until the 70th day of age. Heart rate remained unchanged during all evaluations **(**Fig. 2).

**Table 1:** Characterization of obesity.

Characterization	Control	Obese
Mass (g)	39,78 ± 0,66	38,94 ± 0,74
Lee Index (g/cm³)	0,308 ± 0,002	0,361 ± 0,003****
RP adipose tissue (mg)	149,17 ± 59,97	623,73 ± 17,90****
PG adipose tissue (mg)	190,14 ± 18,20	942,31 ± 85,42****
Abdominal circumference (cm)	9,39 ± 0,11	10,82 ± 0,17****

On the 70^th^ day of life, obesity was characterized by the mass, Lee index, retroperitoneal (RP) and perigonadal (PG) adipose tissue weights and abdominal circumference. Mass was expressed in grams of total weight of mouse (g), Lee index was expressed in grams per cubic centimeter (g/cm³), the weight of RP and PG adipose tissue was expressed in milligrams and the abdominal circumference was expressed in centimeters (cm). Data show mean ± SEM. ****p < 0,0001 when compared control group *vs* obese group. The number of animals was: 22 control and 16 obese mice.

**Figure 3:**
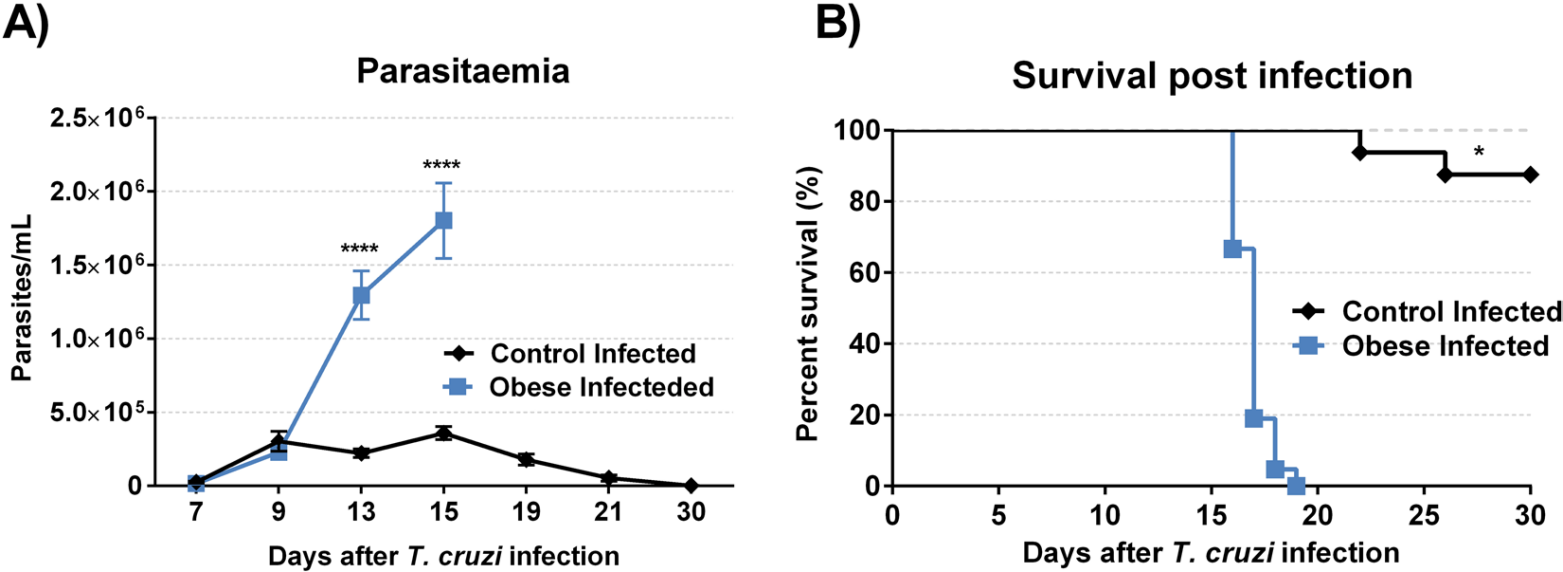
Effect of obesity on time course of *T*. *cruzi* infection in mice. Parasitemia and survival were determined after infection. The parasitaemia **(A)** started on 7^th^ day and ended on 30^th^ day after infection. Data show mean ± SEM. ****p < 0.0001 when compared control infected *vs* obese infected mice. The survival rates **(B)** was evaluated on the same mice used in parasitaemia and was determined by Gehan-Breslow-Wilcoxon test. *p < 0.05 comparing control infected with obese infected group. For this experiment were used 15 mice per group.

On day 7 and 9 after infection, parasitaemia among groups remained similar. From the 13th day after infection, there was a marked increase in parasitaemia in the IOG compared to that in the ICG **(**Fig. 3A**)**. Animals in the IOG began to die on the 16th day after infection, whereas animals in the ICG began to die on the 22nd day post-infection. All infected obese animals died by the 19^th^ day after infection; however, 87.5% of infected control animals remained alive until the end of the mortality assessment period, 30 days post-infection **(**Fig. 3B**)**.

**Figure 4:**
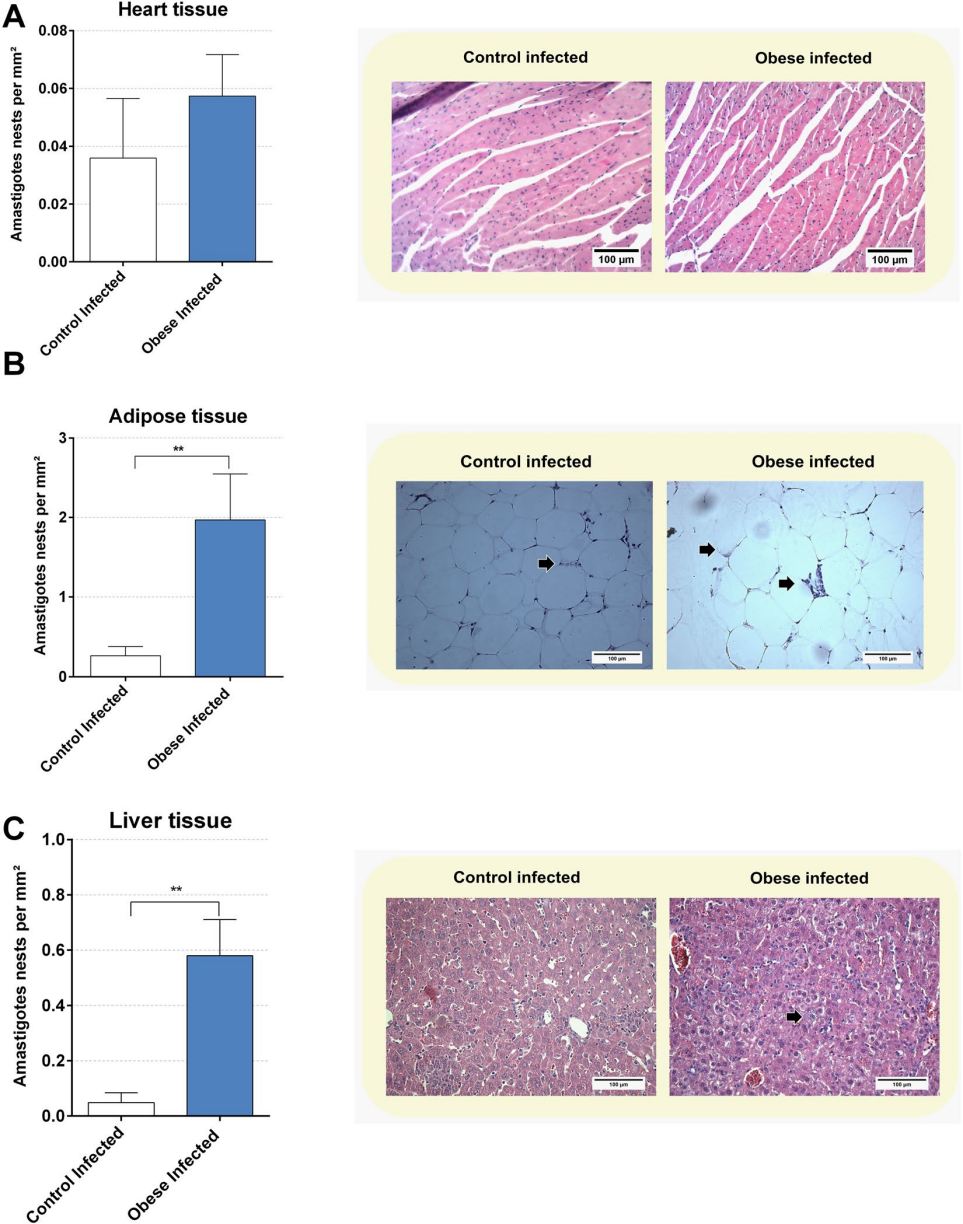
Obesity modulate the tissue parasitism in infected mice. The tissue parasitism was determined in heart tissue **(A)**, retroperitoneal adipose tissue **(B)** and liver tissue **(C)** in 13^th^ day post infection. Representative microphotographs (original magnification X 200) are shown. Black arrows indicate amastigote nests. The results were expressed in numbers of amastigotes nests per square millimeter (mm²). Bars represent mean ± SEM of six mice per group. **p < 0.01 comparing control infected *vs* obese infected. For this experiment we used six animals per group.

Tissue parasitism was assessed by counting the number of amastigote nests in the heart, adipose, and liver tissue. In cardiac tissue **(**Fig. 4A**)**, no statistical difference was found between the ICG and IOG. In retroperitoneal adipose tissue **(**Fig. 4B**)**, an increase in parasitism was found in the IOG compared to that in the ICG (p < 0.01). In the liver tissue **(**Fig. 4C), the IOG had higher parasitism when compared to that in the ICG (p < 0.01).

**Figure 5:**
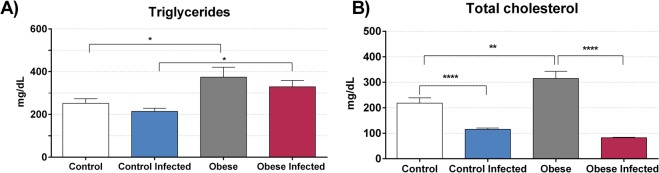
Effect of obesity in lipid profile. The levels of triglycerides **(A**) and total cholesterol **(B)** was determined in serum of mice in 13^th^ day post infection. The results were expressed in milligram per deciliter (mg/dL). *p < 0.05, **p < 0.01 and ****p < 0.0001 when compared between groups. For this experiment, we use eight mice per group.

We then evaluated mouse lipid profiles on day 13 post-infection. Triglyceride levels were increased **(**Fig. 5A**)** in the OG when compared to those in the CG, and when comparing to those in the IOG with those in the ICG (p < 0.05). Infection did not alter plasma triglyceride levels; however, obesity increased plasma total cholesterol concentrations **(**Fig. 5B**)** when compared to those in the CG (p < 0.01). *T*. *cruzi* infection reduced plasma levels of total cholesterol in both the ICG and IOG (p < 0.0001).

**Figure 6:**
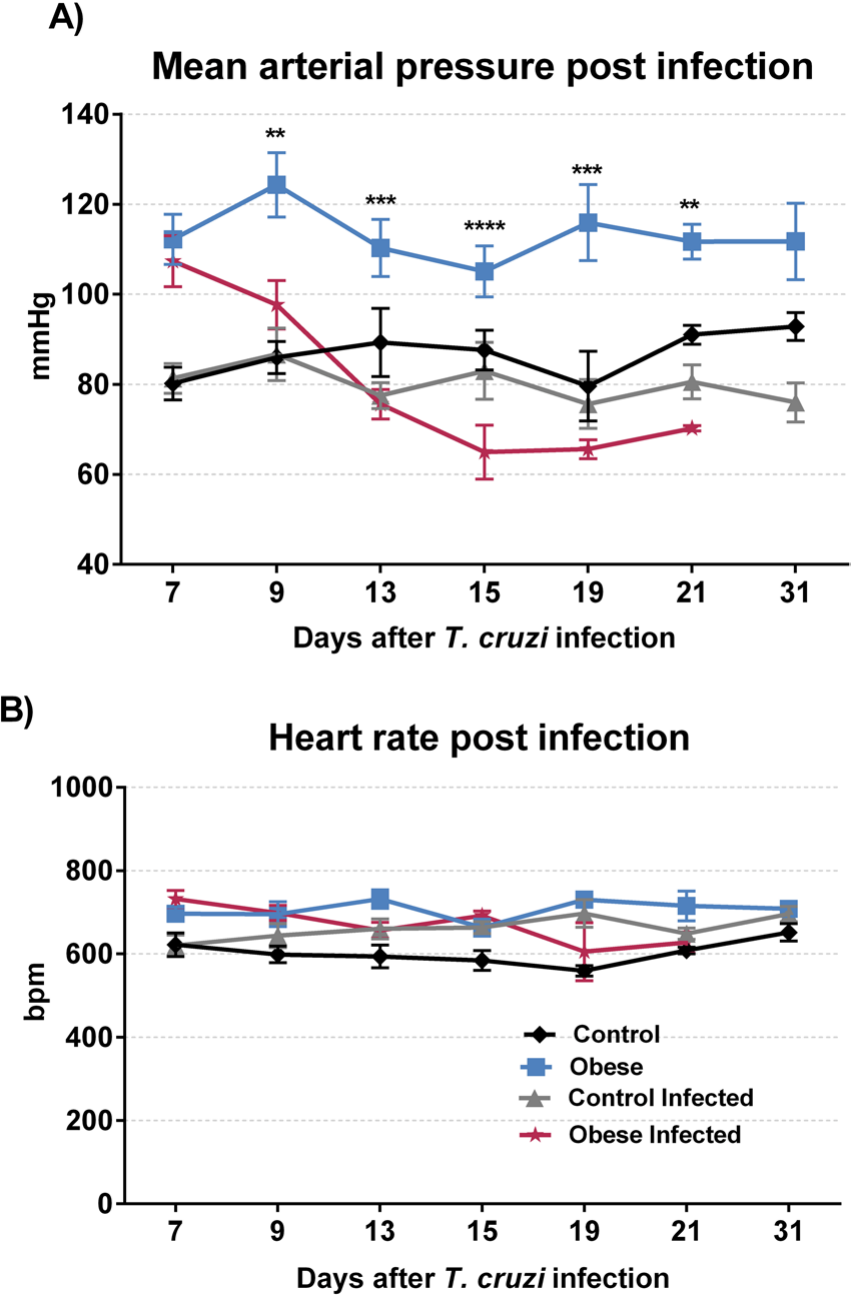
Effect of obesity in cardiovascular parameters during acute phase of *T*. *cruzi* infection. The cardiovascular parameter was measured using the Coda Platform and began in 7^th^ day and finished in 30^th^ day post infection or until death of infected mice. The mean arterial pressure **(A)** was expressed in millimeter of mercury. **p < 0.01, ***p < 0.001 and ****p < 0.0001 when compared obese group vs obese infected group. The heart rate **(B)** was expressed in beats per minute (bpm), no statistical difference was found between the groups. For this experiment was used ten mice per group.

Cardiovascular evaluation after infection was performed on the same days as parasitaemia assessments. A decrease in MAP was observed from the 9^th^ day after infection in the OG and IOG groups (p < 0.01). This difference remained until the 21^st^ day after infection **(**Fig. 6A). No statistical difference was found in MAP values between the ICG and CG groups throughout the evaluation. Moreover, no significant difference was found in heart rate between the IOG and OG groups **(**Fig. 6B).

**Figure 7:**
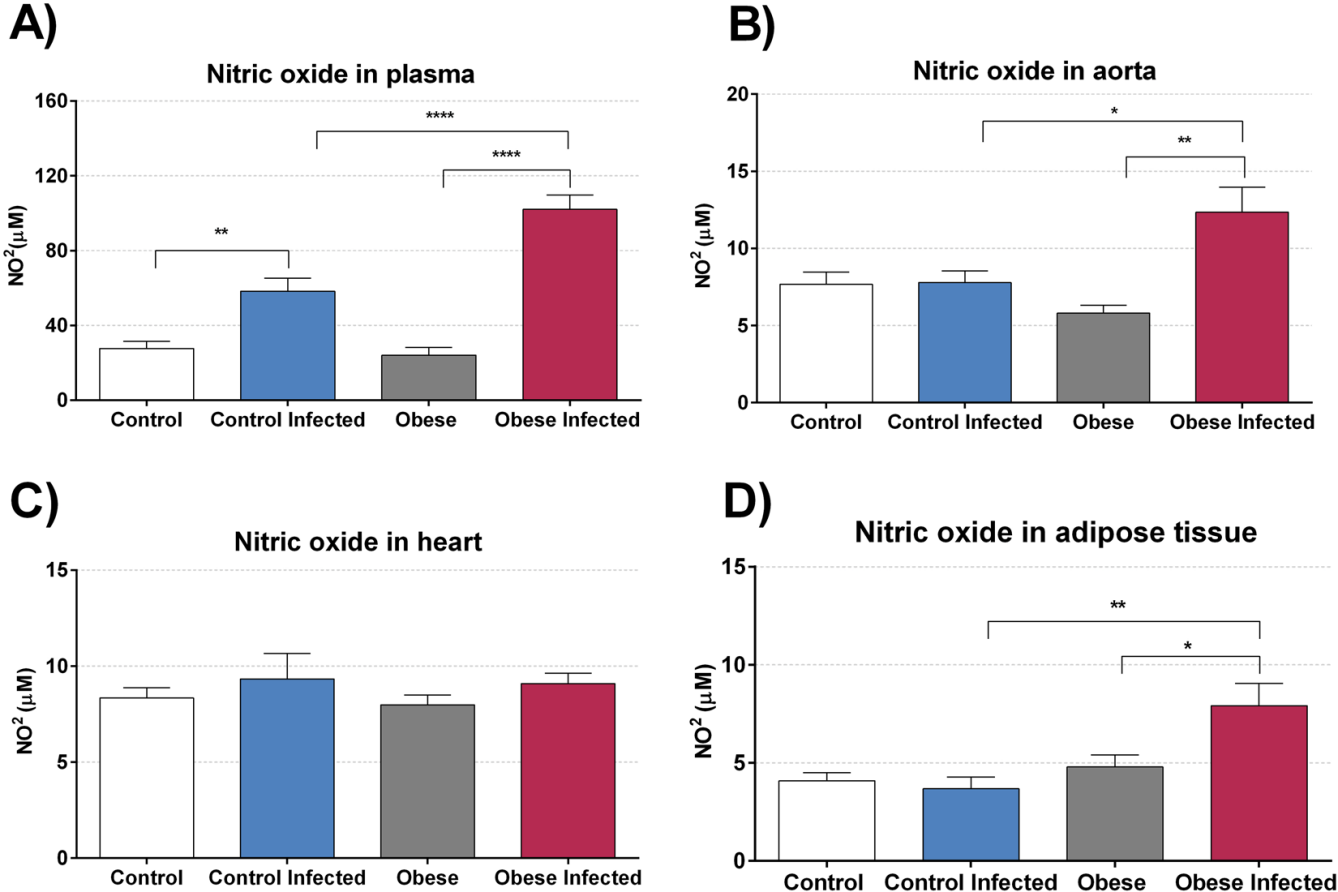
Obesity modulate nitric oxide production. The nitric oxide (NO) was estimated by production of nitrite through cadmium/Griess technique and was measured in plasma (**A**), aorta (**B**), heart tissue (**C**) and adipose tissue from retroperitoneal region (**D**) on 13^th^ day post infection. The levels of NO were expressed in NO micromola**r** (NO^2^µM). Bars represent mean ± SEM of eight mice per group. *p < 0.05, **p < 0.01, ***p < 0.001 and ****p < 0.0001 when compared between groups.

Our results showed an increase in plasma nitrite levels **(**Fig. 7A**)** in the infected animals on the 13^th^ day after infection, both when comparing the CG with the ICG (p < 0.01) and the OG with the IOG (p < 0.0001), with the difference being greater for the latter comparison. The nitrite concentration in the thoracic aorta **(**Fig. 7B**)** was increased only in the IOG when compared to that in the OG (p < 0.001) and also when compared to that in the CG (p < 0.05). Nitrite was also measured in cardiac tissue, but no statistical difference was found between groups **(**Fig. 7C**)**. In adipose tissue **(**Fig. 7D**)**, an increase in nitrite was only found in the IOG when compared to that in the OG (p < 0.05) and the ICG (p < 0.01).

**Figure 8:**
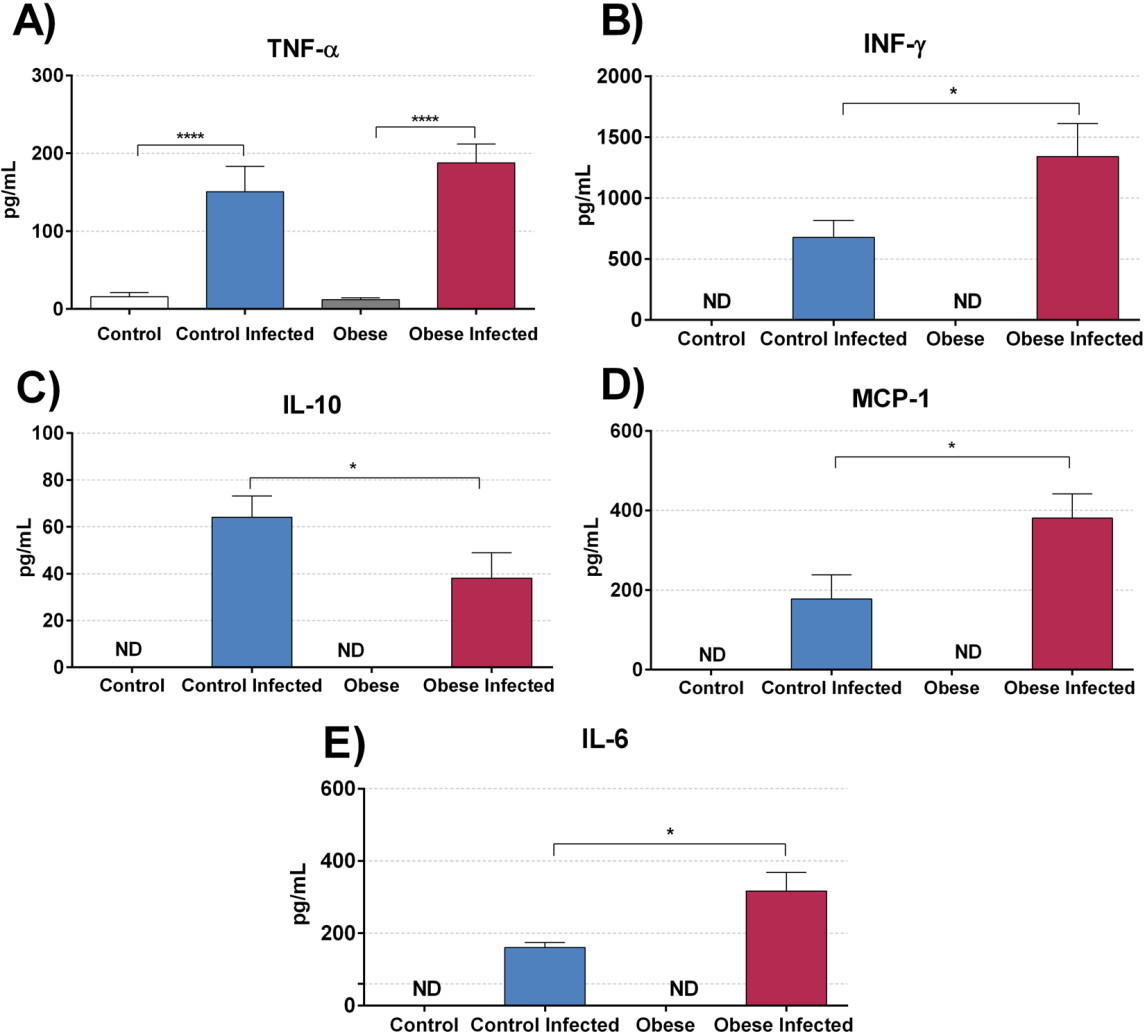
The obesity stimulates production of pro-inflammatory cytokines and decrease anti-inflammatory cytokines in *T*. *cruzi* infection. (**A)** TNF-α, **(B)** INF-γ **(C)** IL-10 **(D)** MCP-1, and **(E)** IL-6 plasma levels were quantified using the BD CBA mouse inflammation kit for a flow cytometer. The data show the mean ± SEM of five mice per group. Bars represent mean ± SEM of eight mice per group. *p < 0.05, ***p < 0.001 and ****p < 0.0001 when compared between groups. (ND) means not identified the cytokines in plasma.

Plasma cytokine levels were determined in all experimental groups. A significant increase in pro-inflammatory cytokines **(**Fig. 8**)**, IFN-γ **(**Fig. 8B**)**, IL-6 **(**Fig. 8E**)**, and MCP-1 **(**Fig. 8E**)** was observed in the plasma of the IOG when compared to that in the ICG (p < 0.05). In contrast, there was a reduction in the anti-inflammatory cytokine IL-10 **(**Fig. 8C**)** in the IOG compared to that in the ICG (p < 0.05).

**Figure 9:**
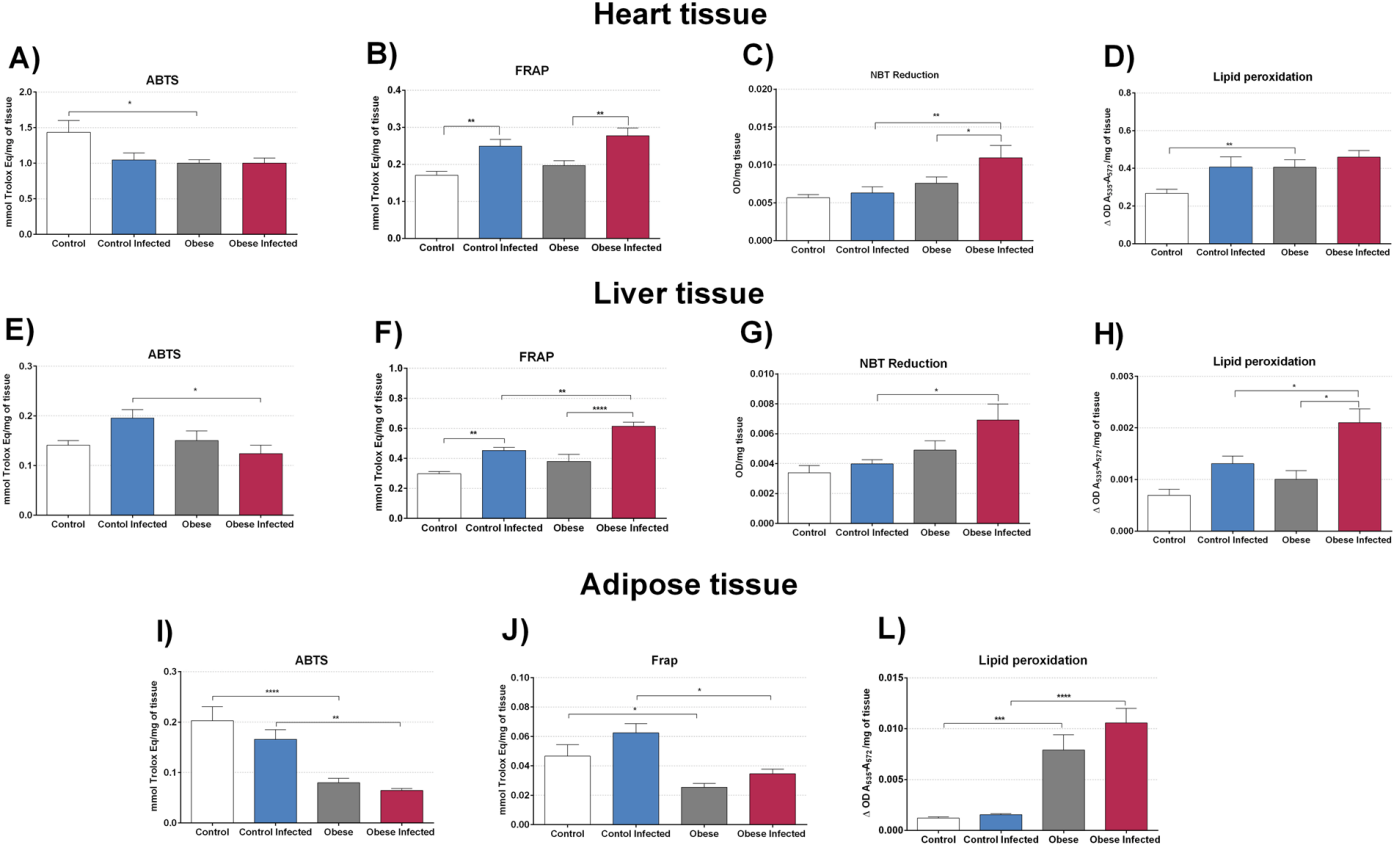
Effect of obesity in oxidative stress. To measure the oxidative stress and anti-oxidant capacity of heart tissue, liver tissue and adipose tissue from retroperitoneal region was used the assay ABTS, which evaluates the total antioxidant capacity, the FRAP assay, which evaluates the total antioxidant power, NBT assay which evaluates the production of the superoxide anion and the evaluation of lipid peroxidation that was determined by the levels of thiobarbituric acid reactive substances (TBARS). We used 6 mice per group. *p < 0.05, **p < 0.01, ***p < 0.001 and ****p < 0.0001.

In the ABTS assay **(**Fig. 9A**)**, a reduction in antioxidant capacity was observed in the OG compared to that in the CG (p < 0.05). An analysis of total antioxidant capacity was also performed using the FRAP assay **(**Fig. 9B**)**, which demonstrated an increase in the hearts of ICG animals when compared to that in the CG (p < 0.01) and in the IOG compared to that in the OG (p < 0.01). An increase in superoxide anion production **(**Fig. 9C**)** was only found in the IOG when compared to that in the ICG (p < 0.01) and OG (p < 0.05). Lipid peroxidation was increased in the OG **(**Fig. 9D**)** when compared to that in the CG (p < 0.01). Infection did not increase lipid peroxidation in the heart in both groups.

Oxidative and antioxidant capacity was also evaluated in the liver. Only the ICG showed an improved ability to sequester the ABTS cation **(**Fig. 9E**)** when compared to that in the IOG (p < 0.05). The total antioxidant capacity obtained based on the FRAP assay **(**Fig. 9F**)** was increased in the infected groups when compared to that in the uninfected groups. In this experiment, we also observed an increase in total antioxidative capacity in the IOG compared to that in the ICG (p < 0.01). Superoxide anion production was evaluated by an NBT assay **(**Fig. 9G, which showed an increase in superoxide anion production in the IOG when compared to that in the ICG (p < 0.05). The TBARS assay **(**Fig. 9H**)** showed an increase only in the IOG when compared to that in the OG (p < 0.05) and ICG (p < 0.05).

A reduction in ABTS antioxidant capacity in the retroperitoneal adipose tissue **(**Fig. 9I**)** of the OG was found when compared to that in the CG, (p < 0.0001), which remained reduced throughout infection when comparing ICG with IOG groups (p < 0.0001). We also found a reduction in total antioxidant capacity based on the FRAP assay **(**Fig. 9J**)** in the OG when compared to that in the CG (p < 0.05) and in the IOG when compared to that in the ICG (p < 0.05). Obesity increased lipid peroxidation in adipose tissue **(**Fig. 9J**)**, when comparing both the OG with the CG (p < 0.001) and the IOG with the ICG (p < 0.0001).

**Figure 10:**
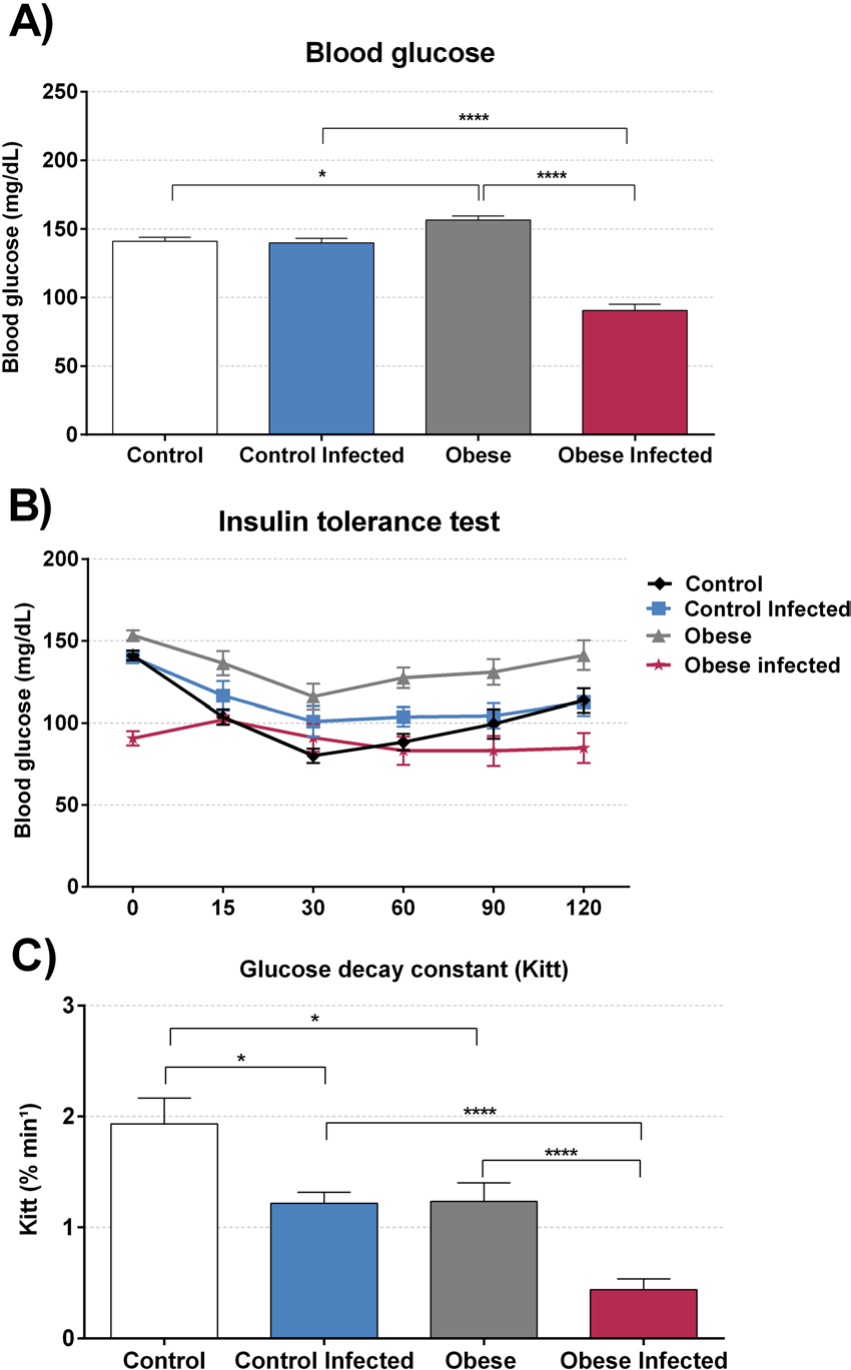
Effect of obesity in glucose metabolism of *T*. *cruzi* infection. The basal blood glucose **(A)** was measured in 2 µL of blood in tail of mice. The blood glucose was expressed in milligram per deciliter (mg/dL), after that was administrate 0,75U/g of insulin, and collected the blood at times 15, 30, 60, 90 and 120 minutes post injection of insulin for performs the insulin test tolerance (ITT) **(B)**, with this measure we calculate the glucose decay constant (K_itt_) **(C)** *p < 0.05, **p < 0.01 and ****p < 0.0001. For this experiment it was utilized nine mice per group.

Fasting glycaemia **(**Fig. 10A**)** was increased in the OG when compared to that in the CG (p < 0.05). However, there was a sharp decline in glycaemic values compared to that in the IOG (p < 0.0001) and the ICG (p < 0.0001). In the ICG, there was no difference between in glycaemic values compared to those in the CG. In addition, an insulin tolerance test **(**Fig. 10B**)** was performed and the Kitt was calculated **(**Fig. 10C**)**. Through this test, we could first observe that obesity alone was able to decrease insulin sensitivity when comparing the CG to the OG (p < 0.05). Infection reduced insulin sensitivity in the IOG when compared to that in the ICG (p < 0.05). Interestingly, in the obese group, *T*. *cruzi* infection intensified insulin resistance, practically without altering the glycaemic level after the application of insulin. The glucose decay constant (kitt) in IOG mice was not statistically different from that in the OG and ICG.

**Figure 11:**
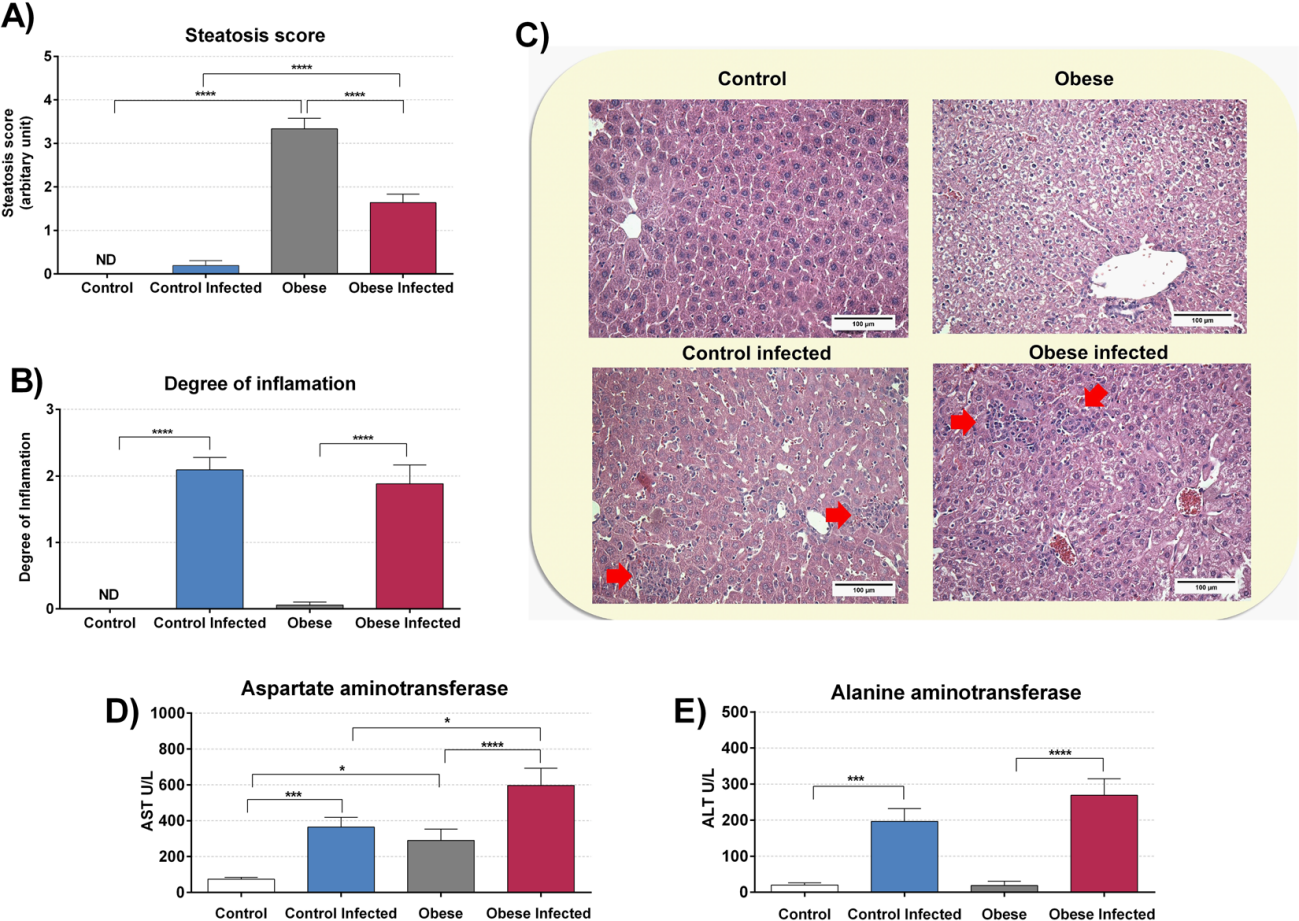
*T*. *cruzi* infection and obesity alters liver tissue. The degree of inflammation **(B)** was graded using a 4-stage classification scale, where 0 = without inflammatory infiltrate and 3 = intense inflammatory infiltrate, in 10 fields per section of tissue (magnification 100×). Hepatic steatosis **(A)** was evaluated in the same 10 fields. To classify the intensity of steatosis a scale with score of 0 to 4 was used. Representative microphotographs **(C)** (original magnification X 200) of liver tissues of mice are shown, red arrows indicates inflammatory infiltrate. For this experiment it was utilized six mice per group. The aspartate aminotransferase (AST) **(D)** and alanine aminotransferase **(E)** (ALT) was evaluated in serum. *p < 0.05, **p < 0.01, ***p < 0.001 and ****p < 0.0001.

Liver histological analyses showed an increase in steatosis score in the OG when compared to that in the CG **(**Fig. 11A**)**. This evaluation consisted of a scale of 0 to 4 where 0 indicated the absence of steatosis and 4 was severe steatosis; in the CG, no steatosis was detected. The mean score in the IOG was 3.33 ± 0.24. After infection, only a few steatotic cells were detected in the liver of the ICG, reaching a mean score of 0.19 ± 0.11. However, infection in the IOG decreased the mean steatosis score when compared to that in the OG (p < 0.0001). Hepatic histological analysis was performed to obtain a score that evaluates hepatic inflammation **(**Fig. 11B**)**. As expected, we observed an increase in the degree of inflammation in the infected animals compared to that in controls without infection.

There was also an increase in AST **(**Fig. 11D**)** when comparing the OG with the CG (p < 0.05) without infection. Infection increased AST levels in both IOG (p < 0.01) and ICG (p < 0.001) groups, and IOG presented higher levels of AST compared to that in the ICG (p < 0.05). As shown in Fig. 11E, we observed an increase in serum ALT levels only in infected animals as compared to those in the uninfected groups (p < 0.0001).

**Figure 12:**
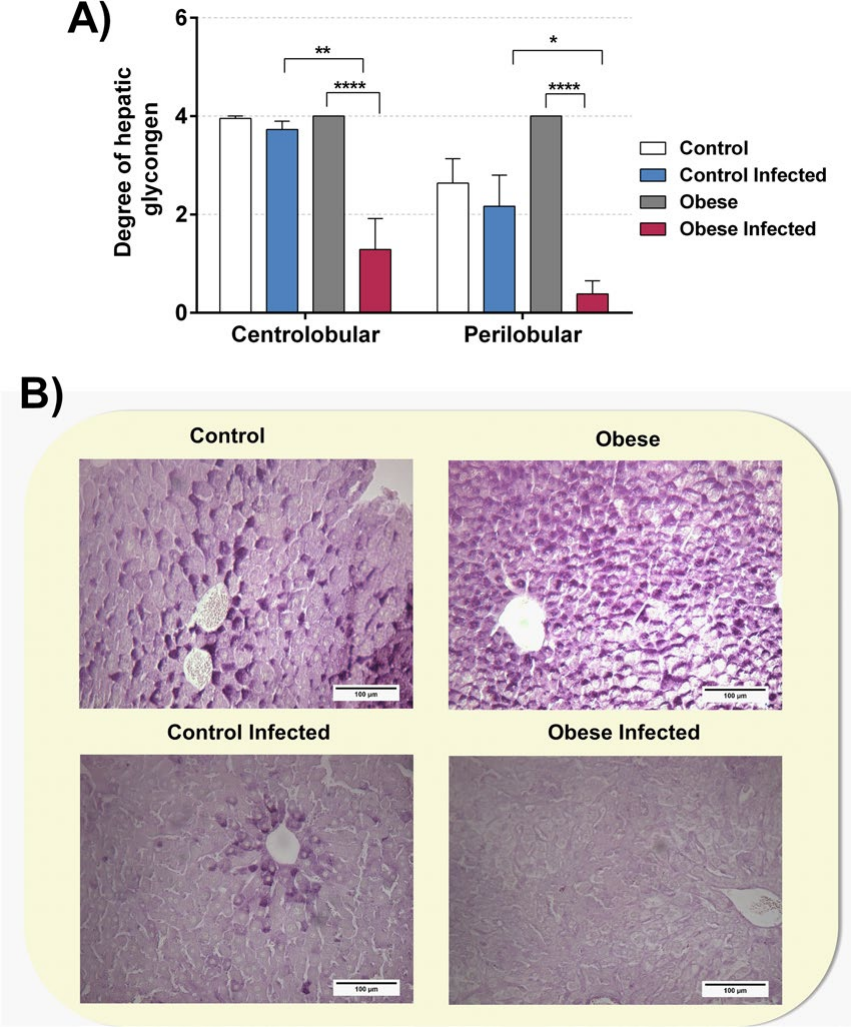
Obese mice infected with *T*. *cruzi* reduces hepatic glycogen. We utilized blades stained in the reaction periodic acid-Schiff for analyze and quantification of hepatic glycogen in mice liver. The degree of hepatic glycogen **(A)** was graded using a positive hepatocyte quantity scale from 0 to 4+, under light microscope with increase in 200×. *p < 0.05, **p < 0.01, ***p < 0.001 and ****p < 0.0001 when compared between groups. Representative photomicrographs **(B)** (original magnification X 200) of liver tissues of mice of different groups are shown. For this experiment it was utilized six mice per group.

An analysis of hepatic glycogen concentrations was performed by PAS. After *T*. *cruzi* infection, a reduction in hepatic glycogen concentration was found in the IOG when compared to that in the OG **(**Fig. 12**)**, both in the centrilobular (p < 0.001) and perilobular region (p < 0.0001). In the CG, infection did not alter the concentration of hepatic glycogen.

## Discussion

The induction of neuroendocrine obesity by neonatal MSG injection proved effective for the development of the MS. Animals submitted to this protocol presented with hypertension, increased deposition of retroperitoneal and perigonadal fat, insulin resistance, and dyslipidaemia, corroborating previous studies^42–48^. MS observed in these mice promoted a higher susceptibility to *T*. *cruzi* infection and pathogenicity, increasing parasitaemia, promoting tissue parasitism, increasing inflammation, and leading to early mortality in these mice. Although we cannot be absolutely certain of the cause of death, we observed that obese animals before death had higher concentrations of NO in plasma and the aorta, which coincided with low MAP values. Animal death could be explained by “circulatory shock” probably due to excess NO, causing systemic vasodilation and decreased peripheral vascular resistance. In addition, the decrease in IL-10 observed can also help to explain animal death, since the genetic inactivation or immunologic neutralisation of IL-10 increases mortality and acute myocarditis in mouse models of *T*. *cruzi* infection^49–51^.

The site of the nervous system most affected by MSG injections in neonate mice is the arcuate nucleus of the hypothalamus, precisely in the region where leptin exerts its main effects to control satiety and food intake^52,53^. In a study by Nagajyothi, *et al*.^54^, which used *db/db* FVB mice characterised by obesity and hyperglycaemia due to the absence of the leptin receptor B isoform (LEPR-B), obese and *T*. *cruzi*-infected mice showed high parasitaemia, intense parasitism, and early death. In our study, using the MSG obesity model, similar findings were observed. Moreover, in the same work by Nagajyothi, *et al*.^54^, LEPR expression only in the central nervous system of NSE-Rb *db/db* mice reduced hyperglycaemia and adiposity, reducing *T*. *cruzi* infection and pathogenicity in these animals. This evidence demonstrates that leptin signalling in the central nervous system is more relevant during *T*. *cruzi* infection when compared to that in the hyperlipidic diet model. In that model of obesity, although the animals presented with higher parasitaemia, there was no early mortality, and in some cases even a protective effect was observed^55,56^.

It is important to note that although similar levels of cardiac parasitism were observed in obese animals compared to that in control animals, the adipose tissue of the OG exhibited higher parasitism. Brima^55^ found similar results wherein excess adipose tissue in the obese mice functioned as a depot, absorbing the trypomastigotes and consequently reducing their presence in the heart. One hypothesis to explain this phenomenon is based on the ability of trypomastigotes to use the low density lipoprotein (LDL) receptor to infect adipocytes^57^. The explanation that can be given for the greater tropism by adipose tissue lies in the greater availability of energetic substrates for *T*. *cruzi* in this tissue. Considering that obese animals have more adipocytes this aspect could explain the greater tropism as well.

Metabolic syndrome is associated with elevated levels of circulating LDL^58^, and this elevation promotes a decrease in the expression of LDL receptors in cells^59^. Although our study only observed a decline in circulating levels of total cholesterol during infection, this suggests that there was also a drop in the circulating levels of LDL in these mice. Consequently, we inferred that this promotes an increase in LDL receptors available for trypomastigotes to infect adipocytes. Strengthening our hypothesis, treatment with atorvastatin in mice submitted to a hyperlipidic diet promotes an increase in mortality and parasitism in adipose and cardiac tissue during the acute phase of *T*. *cruzi* infection^18^, and this phenomenon was explained by the greater presence of free LDL receptors in cardiac and adipose tissue.

The acute phase of infection was found to modulate cardiovascular parameters, mainly in obese mice. In these animals, MAP values decreased from the ninth day of infection, which was accentuated with the progression of infection. This decrease in MAP can be explained by the higher production of NO in plasma.

NO has numerous effects on the body, including its ability to lower blood pressure by promoting the relaxation of vascular smooth muscle, directly affecting peripheral vascular resistance^60^. The expression of iNOS occurs in inflammatory cells present at sites of active inflammation^61^. The acute phase of *T*. *cruzi* infection promotes the enhanced production of proinflammatory cytokines leading to the induction of iNOS expression. NO produced by this pathway has an extremely efficient trypanocidal effect, fundamental for parasitic control in the murine host^33^; however, the hyperproduction of NO by the iNOS pathway can generate serious systemic consequences during *T*. *cruzi* infection. In the work of Santiago^62^, excessive NO production in *phox* KO mice in the acute phase of *T*. *cruzi* infection was associated with an early and permanent decline in MAP, an effect that was reversed by treatment with 1400w, a selective inhibitor of iNOS. A previous study by our research group^27^ also showed a correlation between increased plasma NO production and MAP decreases in mice during the acute phase of *T*. *cruzi* infection.

The adequate production of proinflammatory cytokines such as INF-γ, TNF-α, IL-1, IL-12, and IL-6 is essential for the control of infection by intracellular parasites. Resistance against *T*. *cruzi* infection is mainly related to the ability of lymphocytes to produce INF-^γ^^63^ and TNF-α^9^. These cytokines stimulate macrophages to produce NO, the main effector required for the control of *T*. *cruzi* intracellular multiplication^64^, but its hyperproduction is also related to the severity of *T*. *cruzi* infection, mainly caused by tissue injury^8^. The increased production of proinflammatory cytokines in the obese infected group was related to the early mortality of these animals, suggesting that both NO and proinflammatory cytokines might be involved in infection severity in obese animals. However, neither the increase in proinflammatory cytokines nor NO in obese animals could control parasitaemia and tissue parasitism, in agreement with findings of Brima^55^, Nagajyothi^54^ and Cabalen^56^.

The adipose tissue in obese mice releases increased amounts of proinflammatory cytokines and leptin^65–67^. In monocytes and macrophages, leptin increases the production of proinflammatory cytokines such as TNF-α, IL-6, and IL-12, and stimulates neutrophil activation and monocyte proliferation *in vitro*^68^. However, increased plasma levels of leptin might cause resistance to leptin signalling. In this context, the insensitivity of the leptin receptor could be identified in T cells as a state of leptin deficiency, which results in immune system dysfunction similar to that induced by malnutrition^69^. This could, at least in part, explain the enhanced susceptibility to infection in this model of obesity, even though higher levels of inflammation were observed.

Systemic oxidative stress is an imbalance between the generation of oxidants and antioxidant defence mechanisms, which results in the intracellular accumulation of reactive oxygen species (ROS)^70^. Studies in humans and experimental models of obesity have demonstrated a strong correlation between adiposity and markers of systemic oxidative stress^71^. Our obese animals presented with an imbalance between oxidative status (TBARS and NBT Reduction) and antioxidant capacity (FRAP and ABTS), resulting in oxidative stress when compared to that in the control group, in hepatic, cardiac, and adipose tissue.

In obese animals, adipose tissue is affected by the infiltration of macrophages, and the degree of infiltration correlates positively with body adiposity^71^. Macrophage infiltration is increased in visceral adipose tissue and is directly related to markers of obesity-associated morbidities. The infiltrated macrophages are probably the largest source of ROS and inflammatory cytokines in adipose tissue^70^. The proinflammatory activity of adipokines and the infiltration of macrophages into adipose tissue result in the characterisation of obesity as a low-grade chronic inflammation state and are importantly related to increased insulin resistance, type 2 diabetes, atherosclerosis, and other components of the MS related to inflammation^72^. This might explain the insulin resistance observed in our obese animals, which was aggravated by infection, as there was greater oxidative stress in all tissues assessed. In addition, although in our study we did not evaluate the inflammatory profile of the adipose tissue of infected obese animals, Nagajyothi, *et al*.^73^ demonstrated an increase in the expression of proinflammatory cytokines and chemokines, including IL-1β, IFN-γ, TNF-α, CCL2, CCL5, and CXCL10, as well as an increase in the expression of Toll-like receptors-2 and 9 and activation of the notch pathway, in adipocytes infected by *T*. *cruzi*. Thus, it is very likely that similar changes occurred in our infected obese animals.

The passage of *T*. *cruzi* by the vertebrate host is a challenge for survival. Macrophages are one of the first lines of defence against intracellular pathogen invasion due to their ability to recognise, phagocytise, and destroy microorganisms. Upon penetrating the vertebrate host, *T*. *cruzi* mainly infects phagocytic cells, which respond to invasion by producing ROS like superoxide (O_2_•−) by NADPH oxidase (Nox2), in a process known as respiratory explosion^74–76^. O_2_•− can react with NO resulting from expression of the enzyme iNOS, producing peroxynitrite (ONOO•), NO_2_−, and CO_3_•− with potent oxidative and cytotoxic effects on *T*. *cruzi*, which can also be harmful to the vertebrate host^74,77,78^. To cope with the respiratory burst and adapt to the conditions imposed by its digenetic life cycle, *T*. *cruzi* has efficient and well-regulated antioxidant machinery^79,80^.

Several studies have tried to elucidate the role of ROS during *T*. *cruzi* infection, but the results obtained were often contradictory. Although some studies have suggested that ROS produced during a respiratory burst plays an important role in the control of *T*. *cruzi* infection^74,76,80,81^, other authors have demonstrated that ROS are important for signalling and proliferation in this parasite^82–85^. *T*. *cruzi* is protected from the oxidizing environment by peroxiredoxins (PRX). To establish infection, metacyclic trypomastigotes must invade macrophages and survive the highly oxidative conditions within the phagosome. PRX plays a major role in minimizing the formation of peroxynitrite-derived radicals such as OH, NO_2_− and CO_3_ anions^86^. Velasquez, *et al*.^87^ cloned and sequenced the gene encoding the PRX enzyme in the Y strain of *T*. *cruzi* and confirmed its presence in the mitochondrial compartment. This might be one of the mechanisms responsible for parasite resistance and proliferation. The increase in oxidative stress was unable to neutralise the parasite, probably due to the activity of PRX, which minimises oxidative effects, thus making adipose and hepatic tissue an effective site for its proliferation.

Previous research has shown decreased insulin levels and inadequate responses to glucose metabolism in patients with CD^88,89^. It has also been shown that chemically-induced diabetic mice, as well as genetically predisposed diabetic mice with defective leptin receptors, exhibit increased parasitaemia and mortality after *T*. *cruzi* infection. This suggests that dysregulation of host metabolism might be beneficial for parasitic survival^90^. In our study, the presence of insulin resistance in the obese group seems to have resulted in the early death of these mice.

Prior to infection, obese animals showed glycemia slightly greater than that in the control group. On the 13^th^ day after infection, the obese group was affected by a severe decrease in glucose levels; however, in non-obese animals, no change in glycaemia levels after infection was observed. Hypoglycaemia found in the obese group might have been caused by decreased food intake, excess production of cytokines due to the severity of infection, and/or increased glucose consumption by the parasite, as well as impaired hepatic gluconeogenesis found in the acute phase of CD. Thus, hypoglycaemia has been correlated with the severity of infection and mortality in the acute phase of disease^90,91^. In our study, obese animals had a greater decline in blood glucose and reduced hepatic glycogen levels, and consequently, had the highest mortality rates, a phenomenon that was also observed in other studies^15,54,55^.

*T*. *cruzi* infection in our experimental model promoted a small increase in insulin resistance in the control group and a marked increase in insulin resistance in the obese group. There is still no consensus in the literature regarding the presence or absence of insulin resistance in the acute phase of *T*. *cruzi* infection. In Cabalen’s study^56^ demonstrated an increase in insulin resistance and secretion in the fourth week of infection in the obesity-induced hyperlipidaemic diet group. In the Combs article^15^, *T*. *cruzi* infection, despite promoting a reduction in glucose and insulin levels, did not alter insulin sensitivity during the acute phase, even with high levels of inflammation observed in this study. These discrepancies can be explained by the difference in strains used, as well as the murine model and obesity induction protocol. This increase in insulin resistance found in obese animals might have been due to increased oxidative stress and proinflammatory cytokines found in these mice. In the control mice, the more controlled pro-inflammatory state eventually prevented the establishment of more severe insulin resistance.

The model of obesity used in this study is characterised by the early appearance of non-alcoholic hepatic steatosis, which occurred 70 days post-infection, corroborating the results of Coelho, Franca^92^, which demonstrated the presence of steatosis by the 60^th^ day of age. Accordingly, we found higher plasma levels of AST in the obese group than in the control group.

*T*. *cruzi* infection decreased lipid deposition and reduced hepatic glycogen storage, as well as the decline in glycaemia and total plasma cholesterol levels, in obese animals. This prompted us to hypothesise that *T*. *cruzi* infection causes a decrease in energy reserves available in obese mice. This can be explained by the consumption of host energetic by the parasite, due to its intense state of replication^93^.

In this study we observed a higher parasitic load in the liver of obese animals, and one hypothesis to explain this finding is the high affinity of *T*. *cruzi* for lipids and lipoproteins^94^. As obesity increases, circulating levels of lipids and lipoproteins will also increase in the liver, attracting *T*. *cruzi* to this site^95^. However, increased parasite load in the livers of obese animals did not coincide with a consequent and expected increase in inflammatory infiltrates, since the obese and control groups had similar numbers of cells in the liver. In addition, there were virtually no changes in plasma levels of the hepatic injury markers AST and ALT between the obese and infected control groups, and only AST was slightly higher in the IOG because it was already altered before infection. The work of Lizardo, Almonte^95^ found lower levels of inflammatory infiltrates in the livers of animals submitted to a hyperlipid diet when compared to those in animals that received a regular diet, even though the hyperlipidic diet group presented with greater hepatic parasitism. Even with different experimental designs, these results suggest that obesity might prevent inflammatory infiltration into the liver.

The proinflammatory state, coupled with the dysregulation of metabolism promoted by obesity, seems to be a risk factor during the acute phase of *T*. *cruzi* infection. Not even an increase in NO levels, cytokines, and oxidative stress in plasma and tissues was able to control parasitaemia and parasitism during this phase of infection, resulting in the early mortality of these animals. Thus, the present study demonstrated that MS might be an exacerbating factor for the acute phase of CD. Considering that the incidence of MS is increasing in areas endemic for *T*. *cruzi* infection, this should serve as a warning of the possible associated health risks.

## Supplementary information

Figure 1

## Data Availability

All data generated or analysed during this study are included in this published article (and its Supplementary Information Files).
