# Supplementary material for: Metabolic syndrome agravates cardiovascular, oxidative and inflammatory dysfunction during the acute phase of Trypanosoma cruzi infection in mice
Source: Sci Rep. 2019 Dec 11;9:18885. doi: 10.1038/s41598-019-55363-9 (PMC6906468; doi:10.1038/s41598-019-55363-9)
Supplement: Supplementary file 1 — Figure 1 [file 41598_2019_55363_MOESM1_ESM.pdf]

Metabolic syndrome agravates cardiovascular, oxidative and inflammatory dysfunction during the acute phase of *Trypanosoma cruzi* infection in mice

Bruno Fernando Cruz Lucchetti[1,4], Natalia Boaretto[1], Fernanda Novi Cortegoso Lopes[1], Aparecida Donizette Malvezi[2], Maria Isabel Lovo Martins[2], Vera Lúcia Hideko Tatakihara[2], Victor Fattori[2], Rito Santo Pereira[2], Waldiceu Aparecido Verri Jr[2], Eduardo Jose de Almeida Araujo[3], Phileno Pinge-Filho[2], Marli Cardoso Martins-Pinge[1*]

1 Department of Physiological Sciences, Center of Biological Sciences, State University of Londrina, Londrina - PR, Brazil.

2 Department of Pathological Sciences, Center of Biological Sciences, State University of Londrina, Londrina - PR, Brazil.

3 Department of Histology, Center of Biological Sciences, State University of Londrina, Londrina - PR, Brazil.

4 Department of Physiotherapy, University Center of Araguaia Valley, Barra do Garças – MT, Brazil

*Corresponding author: Marli C. Martins-Pinge, Ph.D.: Departamento de Ciências Fisiológicas, Centro de Ciências Biológicas, Universidade Estadual de Londrina; Rodovia Celso Garcia Cid, Km 380, Campus Universitário, CEP 86055-900, Londrina, PR, Brasil.

Phone.: +55 43 3371 5927

E-mail: martinspinge@uel.br (M.C. Martins-Pinge)

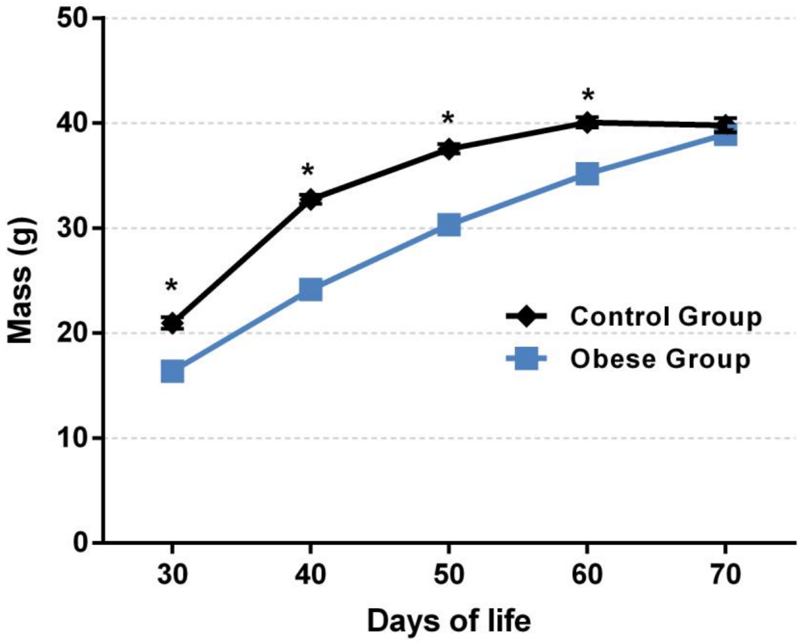

50
40
30
20
10
0
Mass (g)
30
40
50
60
70
Days of life
*
*
*
*
Control Group
Obese Group
